# Use of the Brown-Roberts-Wells Stereotactic Frame in a Developing Country

**DOI:** 10.7759/cureus.2126

**Published:** 2018-01-29

**Authors:** Ali S Khedr, Armando L Alaminos-Bouza, Russell A Brown

**Affiliations:** 1 Neurosurgery, Suez Canal University; 2 Medical Physics, MEVIS Informática Médica Ltda; 3 Principal Engineer, A9.com

**Keywords:** n-localizer, stereotactic surgery planning software, stereotactic neurosurgery, functional neurosurgery, brown-roberts-wells, cosman-roberts-wells, magnetic resonance imaging (mri), computed tomography (ct), positron emission tomography (pet), image guidance

## Abstract

Stereotactic surgery planning software has been created for use with the Brown-Roberts-Wells (BRW) stereotactic frame. This software replaces the Hewlett-Packard calculator originally supplied with the BRW frame and provides modern tools for surgery planning to the BRW frame, which facilitate its potential use as a low-cost alternative to the Cosman-Roberts-Wells (CRW) frame in developing countries.

## Introduction

The Brown-Roberts-Wells (BRW) stereotactic frame [1] was the first clinical stereotactic instrument to incorporate the N-localizer that permits the guidance of stereotactic procedures using medical images obtained via computed tomography (CT), magnetic resonance (MR), or positron emission tomography (PET). Prior to its incorporation into the BRW frame, the N-localizer was validated using a non-clinical, prototype stereotactic frame [2-3]. The N-localizer facilitates the transformation of the $\left ( u_{T}, v_{T} \right )$ pixel coordinates of a target point visualized in a two-dimensional CT, MR, or PET image into the $\left ( x_{T}, y_{T}, z_{T} \right )$ coordinates of that target point defined in the three-dimensional coordinate system of the stereotactic frame [3]. The N-localizer conferred image guidance to the BRW frame via CT or MR, resulting in widespread use of the N-localizer and BRW frame.

**Figure 1:**
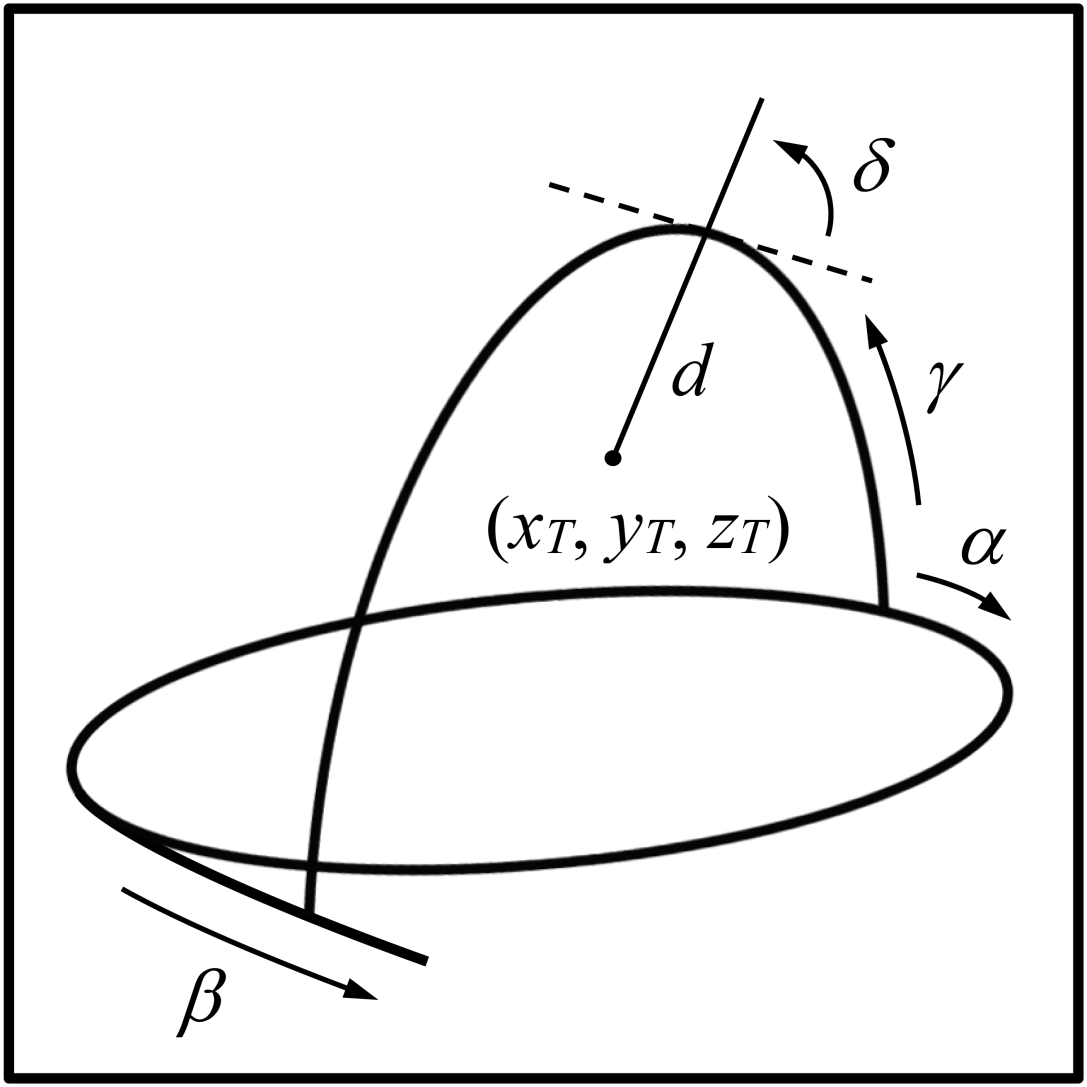
Depiction of the Brown-Roberts-Wells (BRW) stereotactic frame The four angles $\alpha$, $\beta$, $\gamma$, and $\delta$ define a probe insertion trajectory. The probe is inserted to a depth $d$ to reach a target point $\left ( x_{T}, y_{T}, z_{T} \right )$.

The BRW frame defines a probe insertion trajectory via the four angles $\alpha$, $\beta$, $\gamma$, and $\delta$ depicted in Figure 1. In particular, $\alpha$ specifies the azimuth of the point of attachment of the vertical arc to the horizontal ring, $\beta$ specifies the pivot of the arc about its point of attachment to the ring, $\gamma$ specifies the declination of the probe holder along the arc, and $\delta$ specifies the pivot of the probe holder about its point of attachment to the arc. The insertion of a probe to a specified depth $d$ along the trajectory defined by the angles $\alpha$, $\beta$, $\gamma$, and $\delta$ places the tip of the probe at a target point $\left ( x_{T}, y_{T}, z_{T} \right )$. Hence, the $\left ( \alpha, \beta, \gamma, \delta, d \right )$ coordinates specify an intended probe insertion trajectory to a selected target point.

The $\left ( \alpha, \beta, \gamma, \delta, d \right )$ coordinates of the BRW frame may be cumbersome for some surgical procedures because different probe insertion trajectories to the same target point $\left ( x_{T}, y_{T}, z_{T} \right )$ require entirely different $\left ( \alpha, \beta, \gamma, \delta, d \right )$ coordinates for each different trajectory [4]. In contrast to the arc-centered BRW frame, the target-centered Cosman-Roberts-Wells (CRW) frame (Integra LifeSciences Corporation, Plainsboro, NJ) permits more intuitive specification of the probe insertion trajectory [5]. The center of the CRW arc is moved to the target point $\left ( x_{T}, y_{T}, z_{T} \right )$. Then, the declination $\phi$ of the plane of the arc and the angle $\theta$, which is not an azimuth but functions analogously to an azimuth to position the probe holder along the arc, specify the probe insertion trajectory from any direction through roughly a hemisphere centered at $\left ( x_{T}, y_{T}, z_{T} \right )$​​​​​​. Hence, different trajectories to the same target point $\left ( x_{T}, y_{T}, z_{T} \right )$ require only different angles $\phi$ and $\theta$ for each different trajectory. The CRW frame has superseded the BRW frame at many medical centers; nevertheless, the BRW frame remains a viable stereotactic instrument. A used BRW frame is a potentially low-cost alternative to the CRW frame for medical centers that cannot afford a new CRW frame.

## Technical report

A Hewlett-Packard HP-41CV calculator (Palo Alto, California, United States) originally supplied with the BRW frame calculated the $\left ( \alpha, \beta, \gamma, \delta, d \right )$ coordinates. Because a computer is more convenient and powerful than the HP-41CV, stereotactic surgery planning software (SSPS) that is compatible with the CRW frame has been modified by one of us (ALAB) to calculate these coordinates for the BRW frame. The modification of the SSPS was facilitated by the fact that the BRW and CRW frames share a Cartesian coordinate system and several physical components, including the N-localizer assembly.

The calculation of the $\left ( \alpha, \beta, \gamma, \delta, d \right )$ coordinates requires the $\left ( x_{T}, y_{T}, z_{T} \right )$ coordinates of the target point as well as the specification of the probe insertion trajectory. The probe insertion trajectory is specified via the $\left ( x_{E}, y_{E}, z_{E} \right )$ coordinates of an entry point. A vector from the entry point $\left ( x_{E}, y_{E}, z_{E} \right )$ to the target point $\left ( x_{T}, y_{T}, z_{T} \right )$ defines the probe insertion trajectory from which the $\left ( \alpha, \beta, \gamma, \delta, d \right )$ coordinates are calculated [3]. The Appendix presents a novel and robust algorithm for this calculation.

In order to provide a context for the remainder of this article, the four major components of the BRW frame are described as follows: (1) a base ring that is rigidly affixed to the patient's skull, (2) an N-localizer assembly that attaches to the base ring for use during CT scanning, (3) an arc system that attaches to the base ring in place of the N-localizer assembly for use during surgery, and (4) a phantom base to which the arc system attaches prior to surgery for verification of correct $\left ( \alpha, \beta, \gamma, \delta, d \right )$ coordinates.

The following definitions are introduced to promote comprehension of the types of medical images discussed below. Stereotactic and non-stereotactic images are medical images that are obtained with and without the N-localizer assembly affixed to the patient's cranium, respectively. Para-transverse, para-coronal, and para-sagittal images are reconstructed from transverse images but they are not mutually orthogonal transverse, coronal, and sagittal images wherein the coronal and sagittal images are reconstructed from a set of parallel transverse images [6]. Instead, para-transverse, para-coronal, and para-sagittal images are reconstructed from transverse images and may be somewhat oblique to permit the superimposition of vectorial depictions, such as stereotactic atlas data or a probe trajectory on these images (see Figure 3, Figure 4, and Figure 7).

The following functional neurosurgery case illustrates the use of the SSPS with the BRW frame, similar to its use with the CRW frame.

A 60-year-old female was diagnosed with Parkinson's disease six months previously in the neurosurgery clinic at Suez Canal University, Ismailia, Egypt. She presented with hemiparkinsonian symptoms on the left side and exhibited a resting tremor in her left upper and lower extremities, at times persistent and of mild amplitude and at other times intermittently persistent and of moderate amplitude (tremor subscore of 2 for each of the upper and lower extremities), as well as moderate amplitude postural and intention tremor of the left hand (tremor subscore of 3), resulting in a total left-side tremor subscore of 7. She was treated with L-Dopa/Carbidopa 25/200/day. Due to her tremor-predominant Parkinson's disease and her left-side tremor subscore of 7, a thalamotomy was elected.

A few days prior to surgery, a series of transverse, T1-weighted MR images was obtained at 0.9-mm pixel resolution and 1.0-mm spacing between adjacent images. These non-stereotactic MR images demonstrated adequate anatomical detail, particularly for the basal ganglia, ventricles and cortex.

Prior to the fixation of the BRW base ring to the patient's skull, a series of CT images was obtained at 0.4-mm pixel resolution and 0.8-mm spacing between adjacent images. For these non-stereotactic CT images, the CT scanner gantry tilt was 0 degrees to facilitate subsequent 3D image reconstruction.

Immediately following the above-described CT study, the BRW base ring was affixed to the patient's skull and the BRW N-localizer assembly was attached to the base ring. A series of stereotactic CT images was obtained at 0.6-mm pixel resolution and 0.8-mm spacing between adjacent images. Then, the SSPS geometrically aligned the non-stereotactic MR and CT images to the stereotactic CT images using mutual information between the stereotactic and non-stereotactic images [7-8].

**Figure 2:**
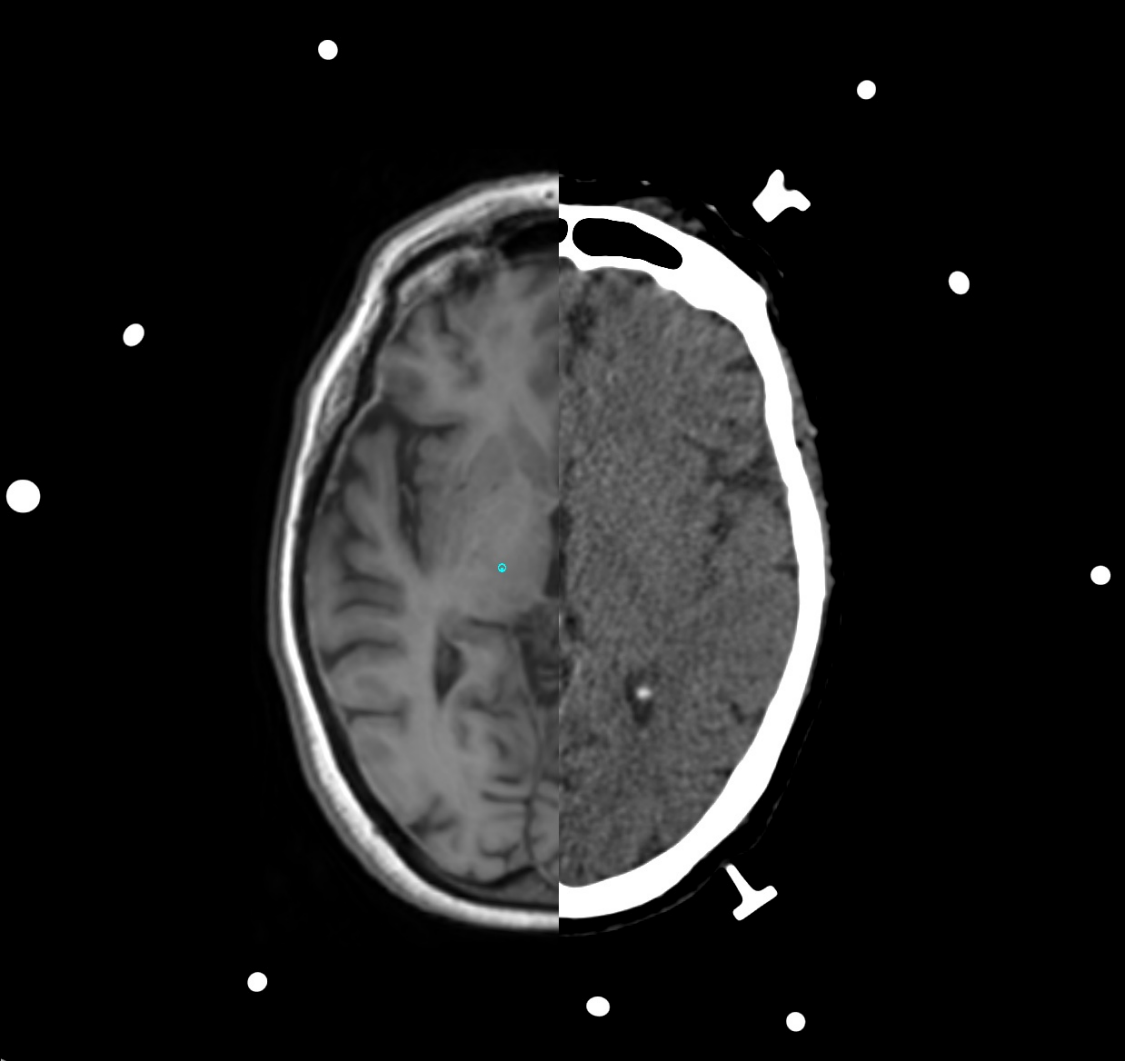
Selection of the target point A target point (depicted by the cyan circle) in the right ventro intermedius nucleus is selected from a non-stereotactic magnetic resonance (MR) image (left) that is geometrically aligned to a stereotactic computed tomography (CT) image (right). Three N-localizers create the circular and elliptical extracranial fiducials.

**Figure 3:**
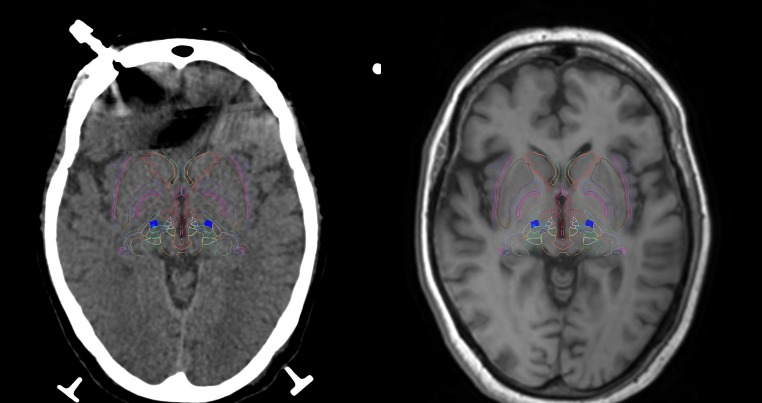
Vectorial depictions of the thalamus and basal ganglia superimposed on para-transverse images Vectorial depictions of the thalamus and basal ganglia (colored lines) are superimposed on para-transverse computed tomography (CT) (left) and magnetic resonance (MR) (right) images. These images are reconstructed from stereotactic CT images and geometrically aligned, non-stereotactic MR images, respectively, in the coordinate system of a vectorial model of the thalamus and basal ganglia that is based on stereotactic atlases [9-10]. The coordinate system of the vectorial model is defined by the anterior commissure (AC), posterior commissure (PC), and a point on the mid-sagittal plane. The model is scaled automatically to match the AC-PC distance prior to superimposition on the para-transverse images and may be rescaled manually thereafter. In each para-transverse image, the target point is depicted by a cyan circle.

**Figure 4:**
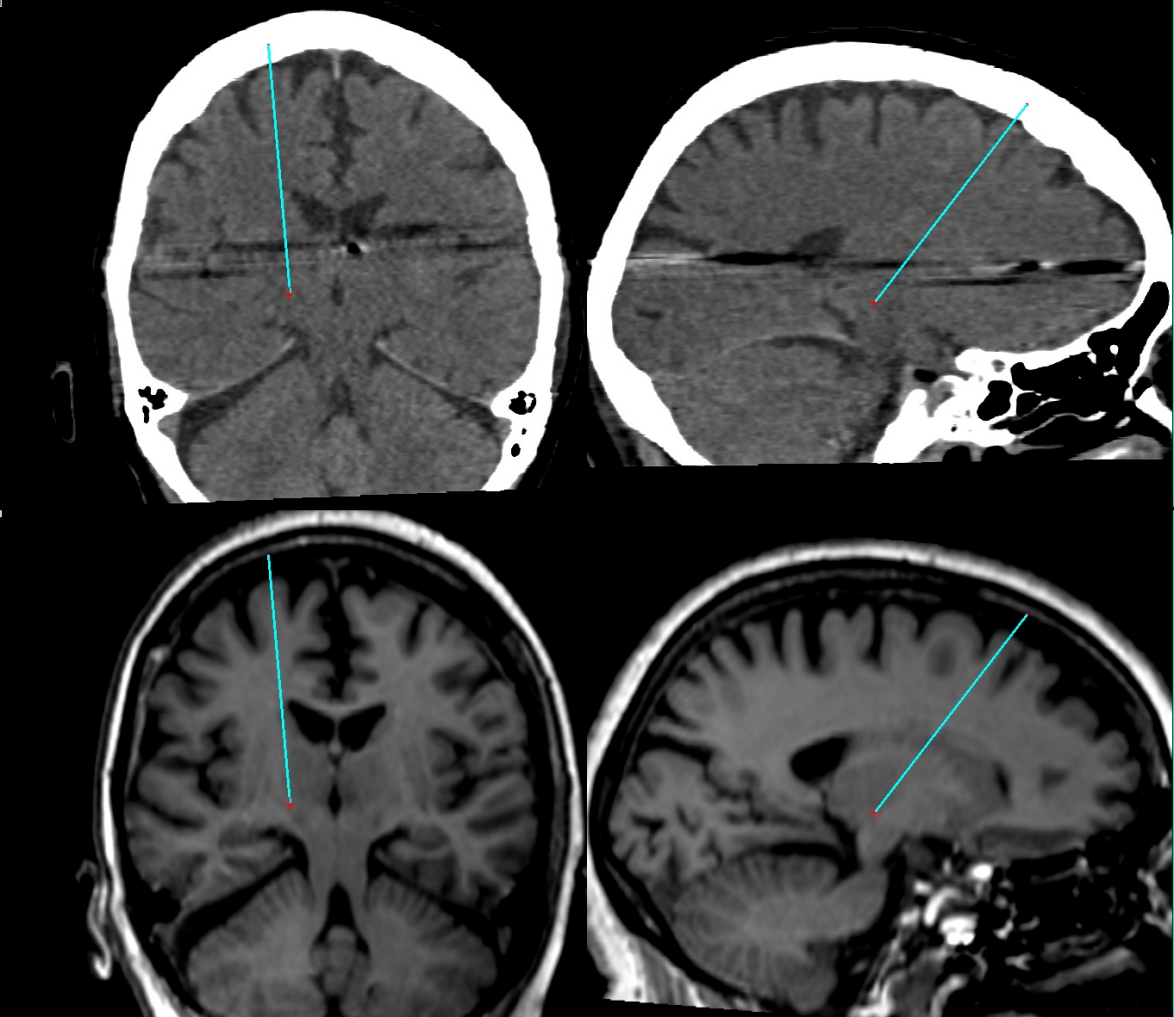
Probe insertion trajectory superimposed on para-coronal and para-sagittal images The target point depicted by the red cross and the entry point define a probe insertion trajectory depicted by the cyan line that is superimposed on para-coronal images (top left and bottom left) and para-sagittal images (top right and bottom right). These images are reconstructed from stereotactic computed tomography (CT) images (top left and top right) and from geometrically aligned, non-stereotactic magnetic resonance (MR) images (bottom left and bottom right). The metal tips of the Brown-Roberts-Wells (BRW) frame fixation screws produce artifacts in the stereotactic CT images. Compare to Figure 7.

The SSPS allowed the selection of a target point in the right ventro intermedius nucleus (Vim) inferred from the border of the thalamic capsule visualized in a geometrically aligned, non-stereotactic MR image (Figure 2). The selection of the target point was aided by comparing the position of the Vim to vectorial depictions of the thalamus and basal ganglia superimposed on para-transverse CT and MR images (Figure 3). The SSPS also allowed the selection of an entry point in a geometrically aligned, non-stereotactic MR image (not shown) so as to define a probe trajectory. Then, the SSPS performed 3D image reconstruction to create orthogonal para-coronal and para-sagittal images containing the probe trajectory to permit verification that the trajectory would avoid the ventricles, sulci, and vascular structures (Figure 4).

After the probe trajectory had been established and verified as discussed above, the target and entry points selected in the geometrically aligned, non-stereotactic MR images were recorded in the corresponding stereotactic CT images to which the MR images had been geometrically aligned. Then, the SSPS transformed the $\left ( u_{T}, v_{T} \right )$ and $\left ( u_{E}, v_{E} \right )$ coordinates of the target and entry points, respectively, from the 2D stereotactic CT images into the $\left ( x_{T}, y_{T}, z_{T} \right )$ and $\left ( x_{E}, y_{E}, z_{E} \right )$ coordinates that defined a probe insertion trajectory in the 3D coordinate system of the BRW frame.

**Figure 5:**
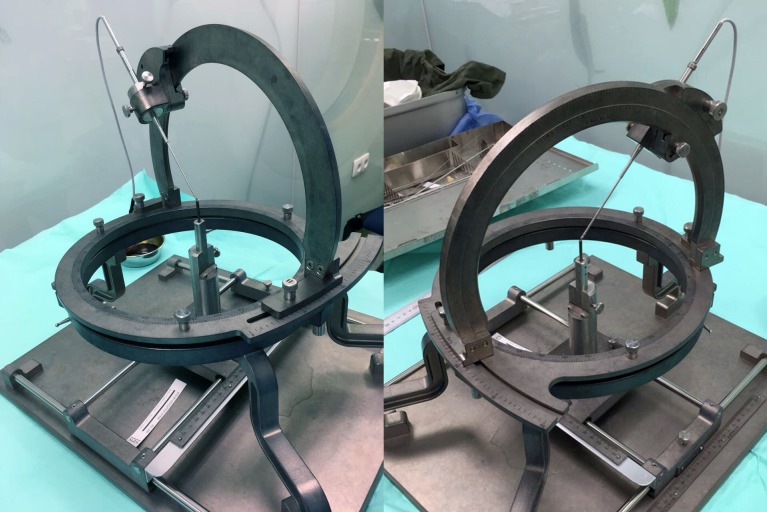
BRW arc system attached to phantom base The phantom base verifies that the calculated $\left ( \alpha, \beta, \gamma, \delta, d \right )$ coordinates direct the tip of the probe to the $\left ( x_{T}, y_{T}, z_{T} \right )$ coordinates of the target point along the probe insertion trajectory. Brown-Roberts-Wells (BRW)

From this probe insertion trajectory, the SSPS calculated the $\left ( \alpha, \beta, \gamma, \delta, d \right )$ coordinates for the BRW frame. The arc system of the BRW frame was attached to the phantom base, the angles $\alpha$, $\beta$, $\gamma$, and $\delta$ were applied to the arc system, and the phantom base provided verification that the tip of a probe inserted to a depth $d$ along the trajectory specified by $\alpha$, $\beta$, $\gamma$, and $\delta$ attained the $\left ( x_{T}, y_{T}, z_{T} \right )$ coordinates of the target point (Figure 5).

Following verification of the probe insertion trajectory, the BRW arc system was transferred to the patient and attached to the base ring. An Inomed TCB013 bipolar, 190-mm long, 2-mm diameter probe (Inomed Medizintechnik GmbH, Emmendingen, Germany) was inserted to the target point. The macrostimulation technique was performed with pulses supplied by an Inomed Neuro N50 lesion generator in order to confirm the target point as a viable site for the thalamotomy. A temporary thermolesion was created by heating to 45C for 30 seconds under the control of the Neuro N50. Following an assessment of the patient's response to this temporary thermolesion, a permanent thermolesion was created by heating to 70C for 60 seconds. The TCB013 probe was withdrawn 2 mm and then the macrostimulation and thermolesion procedure were repeated to create a second temporary thermolesion followed by a permanent thermolesion.

**Figure 6:**
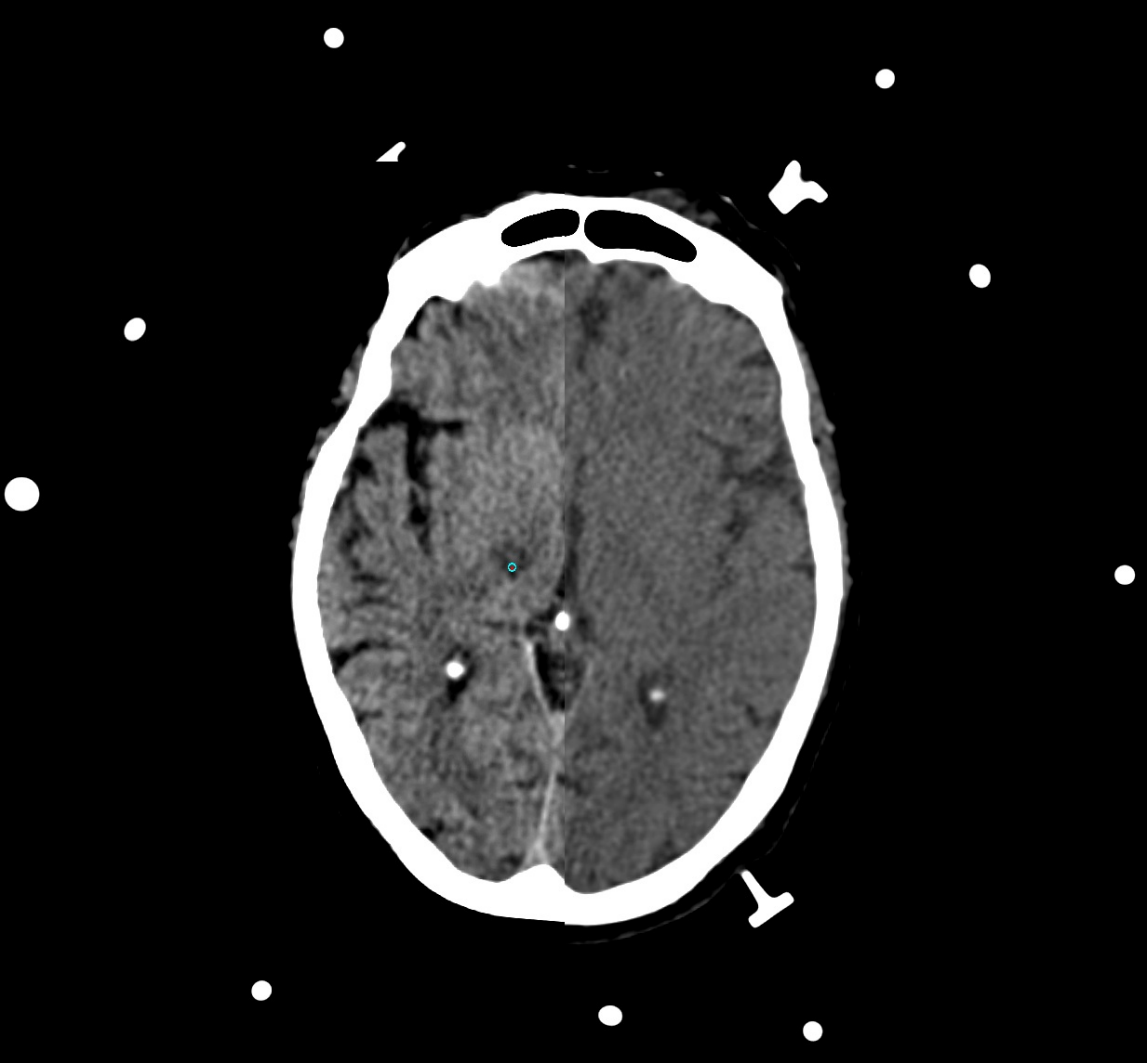
Thalamotomy at the target point The thalamotomy at the right ventro intermedius nucleus is visible as edema surrounding the target point depicted by the cyan circle in a post-operative, non-stereotactic computed tomography (CT) image (left) that is geometrically aligned to a pre-operative, stereotactic CT image (right).

**Figure 7:**
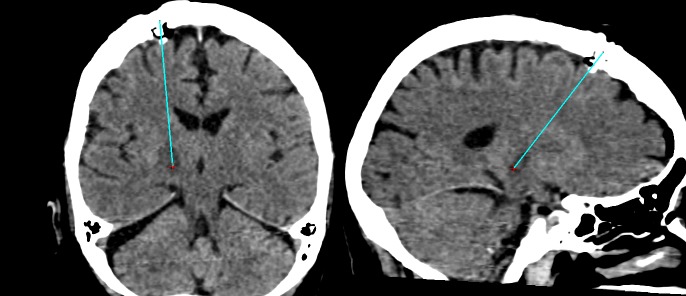
Thalamotomy at the target point and adjacent point The thalamotomy at the right ventro intermedius nucleus is visible as edema surrounding the distal aspect of the probe trajectory depicted by the cyan line in the para-coronal (left) and para-sagittal (right) images. These images are reconstructed from post-operative, non-stereotactic computed tomography (CT) images that are geometrically aligned to pre-operative, stereotactic CT images. The edema is roughly cylindrical because two thermolesions were created 2 mm apart along the probe trajectory. The target point is depicted by the red cross. Compare to Figure 4.

Post-operative transverse, para-coronal, and para-sagittal CT images demonstrated right thalamotomy (Figure 6 and Figure 7). Two weeks following surgery, the patient's total left-side tremor subscore was 0.

## Discussion

Computer technology has progressed considerably during the 39 years that have passed since the introduction of the BRW frame with a Hewlett-Packard HP-41CV calculator. The image processing capabilities made possible by modern computer technology enable stereotactic surgery planning software (SSPS) that integrates information from stereotactic CT images, non-stereotactic CT and MR images, and stereotactic atlases. Notwithstanding the image processing capabilities of the SSPS that can facilitate functional neurosurgery, the electrophysiological confirmation of the target is crucial due to the individual variation of the somatotopy of the patient. For the functional neurosurgery case presented, electrophysiological confirmation was obtained via macrostimulation.

The choice of stereotactic frame for functional neurosurgery is often a matter of personal preference and may be less important than the software used to plan the surgical procedure. The CRW frame is 0.1 mm more accurate than the BRW frame when guided by CT images with slice thicknesses of 1.0 mm and 4.0 mm [11]. However, this difference in mechanical accuracy is less important to the overall accuracy of the stereotactic procedure than is the imaging accuracy, which is strongly influenced by the slice thickness [11-12]. The $\left ( \alpha, \beta, \gamma, \delta, d \right )$ coordinates of the BRW frame that may be cumbersome for some surgical procedures are partially mitigated by the ease of calculation of these coordinates by the SSPS.

## Conclusions

Stereotactic surgery planning software has been adapted to the Brown-Roberts-Wells (BRW) stereotactic frame. This software supersedes the Hewlett-Packard HP-41CV calculator that was supplied with the BRW frame and provides modern tools for surgical planning that facilitate the potential use of the BRW frame as a low-cost alternative to the CRW frame in developing countries.

## Figures and Tables

**Figure 8:**
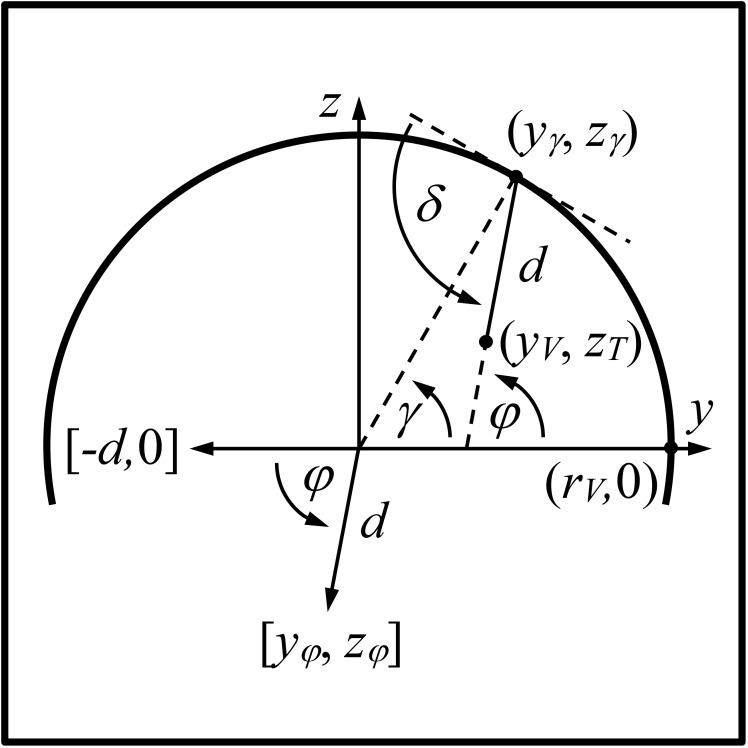
Depiction of the vertical arc of the BRW frame The arc lies in the $yz$-plane when the angles $\alpha$ and $\beta$ both equal zero. The probe insertion depth $d$ and the angles $\gamma$ and $\delta$ determine the $z_{T}$ coordinate of the target point and the intermediate result $y_{V}$. The angle $\varphi$ represents the declination of the probe, where $\varphi = \gamma + \delta - 90$. Adapted from [15]. BRW: Brown-Roberts-Wells

**Figure 9:**
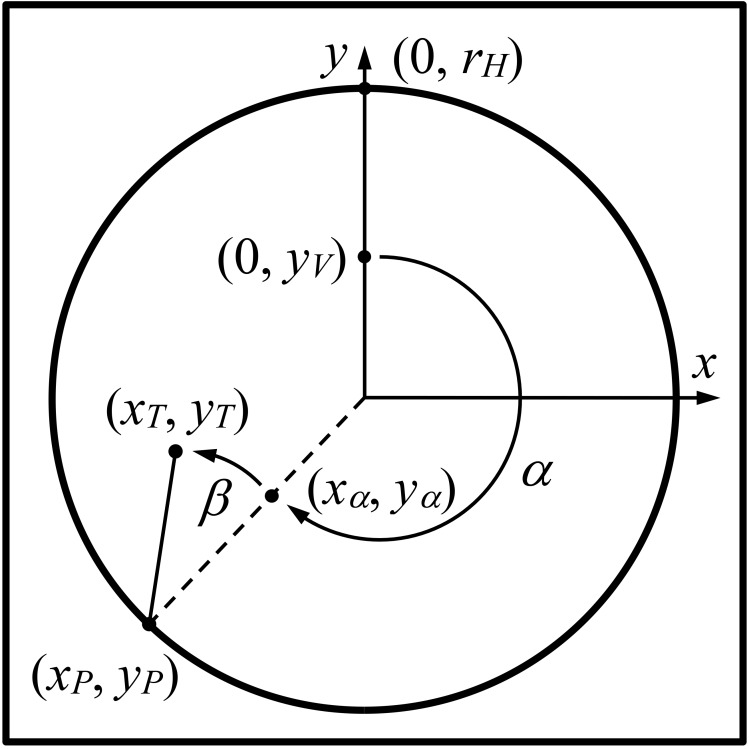
Depiction of the horizontal ring and vertical arc of the BRW frame The ring lies in the $xy$-plane. The arc is rotated out of the $yz$-plane by non-zero values of the angles $\alpha$ and $\beta$. These angles and the intermediate result $y_{V}$ determine the $\left ( x_{T}, y_{T} \right )$ coordinates of the target point. Adapted from [13]. BRW: Brown-Roberts-Wells

## Appendices

The calculation of the $\left ( \alpha, \beta, \gamma, \delta, d \right )$ coordinates requires the $\left ( x_{T}, y_{T}, z_{T} \right )$ coordinates of a target point and the $\left ( x_{E}, y_{E}, z_{E} \right )$ coordinates of an entry point [3]. This calculation is performed by the inverse kinematic function $I$.

$$\left ( \alpha, \beta, \gamma, \delta, d \right ) = I\left ( x_{T}, y_{T}, z_{T}, x_{E}, y_{E}, z_{E} \right )\;\;\;\;\;\;\;\;\;\;\left(1\right)$$

Not all $\left ( \alpha, \beta, \gamma, \delta, d \right )$ coordinates calculated by $I$ can be applied to the BRW frame because the BRW frame restricts the angles $\beta$, $\gamma$, and $\delta$ to certain ranges. Specifically, $\beta$ is limited to the range 0 to 35 degrees, $\gamma$ is limited to the range -5 to 185 degrees, and $\delta$ is limited to the range 35 to 145 degrees. These limitations imply that some calculations produce forbidden $\left ( \alpha, \beta, \gamma, \delta, d \right )$ coordinates that must be rejected by $I$. Previously reported algorithms that calculate $I$ reject some, but not all, forbidden $\left ( \alpha, \beta, \gamma, \delta, d \right )$ coordinates [13-15].

The novel and robust algorithm presented below rejects all forbidden coordinates and uses a forward kinematic function $F$ that calculates the $\left ( x_{T}, y_{T}, z_{T} \right )$ coordinates of a target point from the $\left ( \alpha, \beta, \gamma, \delta, d \right )$ coordinates.

$$\left ( x_{T}, y_{T}, z_{T} \right ) = F\left ( \alpha, \beta, \gamma, \delta, d \right )\;\;\;\;\;\;\;\;\;\;\left(2\right)$$

The function $F$ begins by rotating the point $\left ( 0, r_{V}, 0 \right )$ about the $x$-axis by $\gamma$ degrees to the point $\left (0, y_{\gamma}, z_{\gamma} \right )$, as depicted in Figure 8.

$$\begin{matrix} x_{\gamma} = 0 \\ y_{\gamma} = r_{V} \cos \gamma \\ z_{\gamma} = r_{V} \sin \gamma \end{matrix}\;\;\;\;\;\;\;\;\;\;\left(3\right)$$

where $r_{V}$ represents the radius of the vertical arc

Also, the vector $\left [ 0, -d, 0 \right ]$ is rotated about the $x$-axis by $\varphi$ degrees to the vector $\left [0, y_{\varphi}, z_{\varphi} \right ]$.

$$\begin{matrix} x_{\varphi} = 0 \\ y_{\varphi} = -d \cos \varphi = -d \cos \left ( \gamma + \delta - 90 \right ) = -d \left [ \cos \left ( \gamma + \delta \right ) \cos \left ( -90 \right ) - \sin \left ( \gamma + \delta \right ) \sin \left ( -90 \right ) \right ] = -d \sin \left ( \gamma + \delta \right ) \\ z_{\varphi} = -d \sin \varphi = -d \sin \left ( \gamma + \delta - 90 \right ) = -d \left [ \sin \left ( \gamma + \delta \right ) \cos \left ( -90 \right ) + \cos \left ( \gamma + \delta \right ) \sin \left ( -90 \right ) \right ] = d \cos \left ( \gamma + \delta \right ) \end{matrix}\;\;\;\;\;\;\;\;\;\;\left(4\right)$$

where $\varphi$ represents the declination of the probe [15] and $\varphi = \gamma + \delta - 90$ because when $\delta = 90$ degrees, the probe points toward the origin of the vertical arc.

Adding the vector $\left [0, y_{\varphi}, z_{\varphi} \right ]$ to the point $\left (0, y_{\gamma}, z_{\gamma} \right )$ moves the tip of the probe by the insertion depth $d$ to the point $\left (0, y_{V}, z_{T} \right )$.

$$\begin{matrix} x_{V} = 0 \\ y_{V} = r_{V} \cos \gamma - d \sin \left ( \gamma + \delta \right ) \\ z_{T} = r_{V} \sin \gamma + d \cos \left ( \gamma + \delta \right ) \end{matrix}\;\;\;\;\;\;\;\;\;\;\left(5\right)$$

where $z_{T}$ is the $z$-coordinate of the target point and $y_{V}$ is an intermediate result to be used in subsequent calculations

Next, the point $\left ( 0, y_{V}, z_{T} \right )$ is rotated about the $z$-axis by $-\alpha$ degrees (for the BRW frame, $+\alpha$ describes a clockwise rotation) to the point $\left (x_{\alpha}, y_{\alpha}, z_{T} \right )$, as depicted in Figure 9.

$$\begin{matrix} x_{\alpha} = y_{V} \sin \alpha = \left [ r_{V} \cos \gamma - d \sin \left ( \gamma + \delta \right ) \right ] \sin \alpha \\ y_{\alpha} = y_{V} \cos \alpha = \left [ r_{V} \cos \gamma - d \sin \left ( \gamma + \delta \right ) \right ] \cos \alpha \\ z_{T} = r_{V} \sin \gamma + d \cos \left ( \gamma + \delta \right ) \end{matrix}\;\;\;\;\;\;\;\;\;\;\left(6\right)$$

Similarly, the point $\left ( 0, r_{H}, 0 \right )$ is rotated about the $z$-axis by $-\alpha$ degrees to the point of attachment $\left (x_{P}, y_{P}, 0 \right )$ of the vertical arc to the horizontal ring.

$$\begin{matrix} x_{P} = r_{H} \sin \alpha \\ y_{P} = r_{H} \cos \alpha \\ z_{P} = 0 \end{matrix}\;\;\;\;\;\;\;\;\;\;\left(7\right)$$

where $r_{H}$ represents the radius of the horizontal ring

Finally, the point $\left (x_{\alpha}, y_{\alpha}, z_{T} \right )$ is rotated by $\beta$ degrees about a vector, which is parallel to the $z$-axis and passes through the point $\left (x_{P}, y_{P}, 0 \right )$, to the target point $\left (x_{T}, y_{T}, z_{T} \right )$.

$$\begin{matrix} x_{T} = x_{P} + \left (x_{\alpha} - x_{P} \right ) \cos \beta - \left (y_{\alpha} - y_{P} \right ) \sin \beta \\ y_{T} = y_{P} + \left (x_{\alpha} - x_{P} \right ) \sin \beta + \left (y_{\alpha} - y_{P} \right ) \cos \beta \\ z_{T} = r_{V} \sin \gamma + d \cos \left ( \gamma + \delta \right ) \end{matrix}\;\;\;\;\;\;\;\;\;\;\left(8\right)$$

The forward kinematic function $F$ is used in an iterative search for the $\left ( \alpha, \beta, \gamma, \delta, d \right )$ coordinates that direct a probe to a specific target point $\left ( x_{T}, y_{T}, z_{T} \right )$ along a probe insertion trajectory that passes through a specific entry point $\left ( x_{E}, y_{E}, z_{E} \right )$. This search is facilitated by expressing the vector from the target point to the entry point in terms of spherical coordinates that include an azimuth $\theta_{E}$ and a declination $\phi_{E}$. It is convenient to express this vector in terms of the angles $\theta_{E}$ and $\phi_{E}$ because, for purposes of search, the BRW frame is modeled as a robotic arm wherein each of the angles $\alpha$, $\beta$, $\gamma$, and $\delta$ is considered to be a joint in the arm. The search uses this angular model of the BRW frame to impose the following constraints on the angles via previously published techniques for a constrained search that are typically applied to robotic arms [16].

$$\begin{matrix} \theta = \theta_{E} \\ \phi = \phi_{E} \\ 0 \leq \beta \leq 35 \\ -5 &lt; \gamma \leq 185 \\ 35 \leq \delta \leq 145 \end{matrix}\;\;\;\;\;\;\;\;\;\;\left(9\right)$$

No constraint is imposed on the angle $\alpha$ because it may assume any value from 0 to 360 degrees.
